# Use of Rapid Antigen Testing for SARS-CoV-2 in Remote Communities — Yukon-Kuskokwim Delta Region, Alaska, September 15, 2020–March 1, 2021

**DOI:** 10.15585/mmwr.mm7033a3

**Published:** 2021-08-20

**Authors:** Ellen Hodges, Brian Lefferts, Elizabeth Bates, Christine Desnoyers, Dana Bruden, Michael Bruce, Joseph McLaughlin

**Affiliations:** ^1^Yukon Kuskokwim Health Corporation, Bethel, Alaska; ^2^Arctic Investigations Program, Division of Preparedness and Emerging Infections, National Center for Emerging and Zoonotic Diseases, CDC; ^3^Alaska Department of Health and Social Services.

Controlling the spread of SARS-CoV-2, the virus that causes COVID-19, in Alaska is challenging. Alaska includes many remote and isolated villages with small populations (ranging from 15 to >1,000 persons) that are accessible only by air from larger communities. Until rapid point-of-care testing became widely available, a primary challenge in the diagnosis of COVID-19 in rural Alaska was slow turnaround times for SARS-CoV-2 test results, attributable to the need to transport specimens to testing facilities. To provide more timely test results and isolation of cases, the Yukon Kuskokwim Health Corporation (YKHC) introduced Abbott BinaxNOW COVID-19 Ag rapid antigen test (BinaxNOW) on November 9, 2020, in the rural Yukon-Kuskokwim Delta region in southwestern Alaska. To evaluate the impact of implementing antigen testing, YKHC reviewed the results of 54,981 antigen and molecular tests for SARS-CoV-2 performed in the Yukon-Kuskokwim Delta during September 15, 2020–March 1, 2021. Introduction of rapid, point-of-care testing was followed by a more than threefold reduction in daily SARS-CoV-2 case rates during approximately 1 month before the introduction of COVID-19 vaccination. The median turnaround time for SARS-CoV-2 test results decreased by >30%, from 6.4 days during September 15–November 8, 2020, to 4.4 days during November 9, 2020–March 1, 2021 (p<0.001). Daily incidence decreased 65% after the introduction of BinaxNOW, from 342 cases per 100,000 population during the week of November 9 to 119 during the week of December 13 (p<0.001). These findings indicate that point-of-care rapid antigen testing can be a valuable tool in reducing turnaround times in rural communities where local access to laboratory-based nucleic acid amplification testing (NAAT) is not readily available and could thereby reduce transmission by facilitating rapid isolation of infected persons, contact tracing, and implementation of local mitigation strategies.

YKHC introduced BinaxNOW in Yukon-Kuskokwim Delta villages on November 9, 2020. BinaxNOW is a lateral flow immunoassay performed as a point-of-care test using a nasal swab, with results available in ≤15 minutes. Before the use of BinaxNOW tests, the only local testing capacity in the region was GeneXpert Express (Cepheid) SARS-CoV-2 real-time reverse transcription–polymerase chain reaction (RT-PCR) testing and Abbott ID Now COVID-19 isothermal NAAT in Bethel, Alaska. All other specimens were analyzed by RT-PCR through the Alaska state public health laboratories located in Anchorage and Fairbanks or the Alaska Native Tribal Health Consortium in Anchorage or were sent to a private out-of-state laboratory. Anchorage and Fairbanks are 397 and 522 miles, respectively, from Bethel and are accessible only by air.

Data on persons for whom SARS-CoV-2 antigen or molecular tests were performed in the Yukon-Kuskokwim Delta were obtained from electronic health records at YKHC; 54,981 records of tests performed during September 15, 2020–March 1, 2021 were reviewed. The interval from the date of specimen collection to the date of test result was used to calculate the turnaround time before (September 15–November 8, 2020) and after (November 9, 2020–March 1, 2021) introduction of BinaxNOW testing. During the period after BinaxNOW introduction, the turnaround time was calculated for NAAT and antigen testing combined. The difference in turnaround times between periods was assessed using the Wilcoxon rank-sum test. BinaxNOW testing was performed primarily on symptomatic persons or persons identified as close contacts of persons with a confirmed case of COVID-19.* The daily COVID-19 incidence (cases per 100,000 persons) was assessed, and using Poisson regression, rates were compared before and after introduction of the BinaxNOW testing. SAS (version 9.4; SAS Institute) was used to conduct analyses. Some persons who received a negative test result might have had multiple specimens collected for testing during the study period; however, these retests were not included in the regression analysis. This activity was reviewed by CDC and was conducted consistent with applicable federal law and CDC policy.^†^

Among 16,027 tests for SARS-CoV-2 performed via NAAT during September 15, 2020–November 8, 2020 (before BinaxNOW introduction), 1,223 (7.6%) were positive, 14,778 (92.2%) were negative, and the results for 26 (0.2%) were unknown (Table). The average turnaround time in the central hub community of Bethel was 5.7 days (range = 0.7–13.3 days), and in villages outside Bethel was 7.9 days (range = 0.8–14.6 days). The daily incidence during this time was 342 cases per 100,000 population.

**TABLE:** Number of SARS-CoV-2 tests performed and test results, by test type, and average turnaround time for receipt of test results before and after introduction of Abbott BinaxNOW COVID-19 Ag rapid antigen test — Yukon-Kuskokwim Delta region, Alaska, September 15, 2020–March 1, 2021

Time	Type of test	No. of tests	Test results, no. (%)			Average turnaround time
			Positive	Negative	Unknown	
Before BinaxNow introduction (Sep 15–Nov 8, 2020)	NAAT	16,027	1,223 (7.6)	14,778 (92.2)	26 (0.2)	5.7–7.9 days
After BinaxNow introduction (Nov 9, 2020–Mar 1, 2021)	NAAT	29,835	2,273 (7.6)	27,024 (90.6)	538 (1.8)	7 days
	BinaxNOW	9,119	1,337 (14.7)	7,782 (85.3)	0 (—)	15 mins
	All	38,954	3,610 (9.3)	34,806 (89.3)	538 (1.4)	4.4 days

**Abbreviations:** BinaxNOW = Abbott BinaxNOW COVID-19 Ag rapid antigen test; NAAT = nucleic acid amplification test.

During the period after introduction of BinaxNOW, November 9 through March 1, 2021, among 38,954 total tests performed including 9,119 (23.4%) using BinaxNOW and 29,835 (76.6%) using NAAT, a total of 3,610 (9.3%) were positive, 34,806 (89.4%) were negative, and the results for 538 (1.4%) were unknown. Among the 9,119 BinaxNOW tests, 1,337 (14.7%) were positive, and among 29,835 NAAT 2,273 (7.6%) were positive. BinaxNOW tests accounted for 37% of all positive test results during this period.

The median turnaround times for BinaxNOW and NAAT results were 15 minutes and 7 days, respectively; the overall median turnaround time after BinaxNOW introduction decreased to 4.4 days (range = 0.1–12.4 days) (p<0.001). Daily COVID-19 incidence declined to 119 during the week of December 13, 2020 (p<0.001) (Figure).

**FIGURE:**
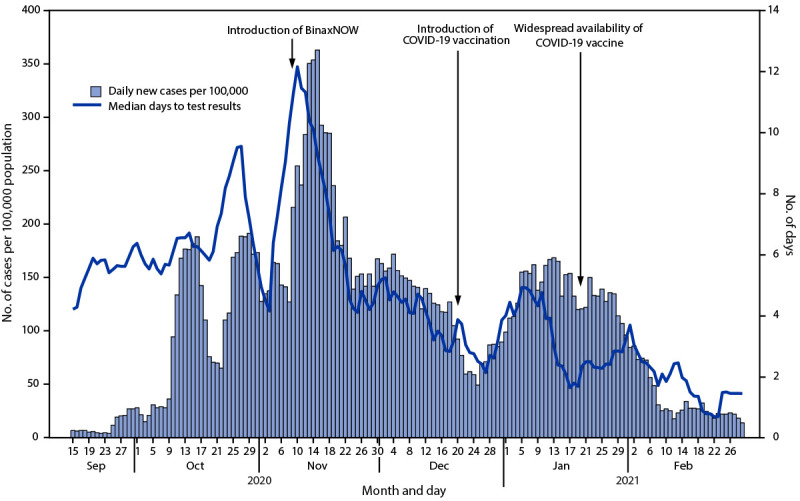
Daily COVID-19 incidence* and median turnaround time for test results, by week — Yukon-Kuskokwim Delta region, Alaska, September 15, 2020–March 1, 2021 **Abbreviation:** BinaxNOW = Abbott BinaxNOW COVID-19 Ag rapid antigen test. * Cases per 100,000 population.

## Discussion

The findings from this ecologic study indicate that introduction of rapid point-of-care antigen testing in remote villages and other community settings allowed for same-day identification of infected persons, which in turn facilitated prompt isolation, contact tracing, and quarantine of close contacts. The use of rapid antigen testing also allowed for implementation of early public health interventions that might have changed the trajectory of SARS-CoV-2 outbreaks occurring in the region, such as recommending specific and tailored prevention strategies to small communities. As cases were identified, local tribal councils and governments were notified to provide situational awareness and prompt appropriate mitigation measures, including scheduled visitation with selected family members for funerals or religious ceremonies and implementation of changes in operations at local businesses to limit capacity. 

The rapid identification and isolation of infected persons made possible by introduction of BinaxNOW testing in early November 2020 might have contributed to a decrease in SARS-CoV-2 transmission before the introduction COVID-19 vaccination in the region on December 17, 2020, and its availability to all Yukon-Kuskokwim Delta residents on January 19, 2021. The sharp drop in cases after February 1 was likely attributable primarily to rapidly increasing vaccination coverage rates, highlighting the importance of vaccination in preventing ongoing transmission of SARS-CoV-2. Rapid identification of cases of COVID-19 after introduction of BinaxNOW had the benefit of promptly providing infected persons with a recovery kit (equipment for home monitoring of temperature and oxygen saturation). Recovery kits were given to infected persons by the health care provider in the village who performed the test. Infected persons at higher risk for complications might have been identified sooner in the course of their illness, allowing for timely monitoring and intervention if their health status began to deteriorate.

The higher percentage of positive test results observed for BinaxNOW testing (14.7%) compared with that for NAAT (7.4%) likely occurred because the antigen tests were used for diagnostic testing of symptomatic persons and close contacts of confirmed cases, whereas the NAAT tests were usually used for screening testing. The difference in percentage of positive test results between BinaxNOW and NAAT might be due in part to the differences in sensitivity and specificity of the two types of tests. The practice of using antigen tests for symptomatic persons and those considered to be at high risk because of exposure and reserving NAAT for those at lower risk might be useful in settings in which resources are limited for NAAT tests or turnaround times are too long to implement effective isolation. Furthermore, rapid identification of persons who are contagious might have important public health implications in regions like the Yukon-Kuskokwim Delta where crowded housing and inadequate sanitation are common (*1*–*3*) and could facilitate more rapid outbreak spread. Further public health impacts in the region include the disproportionate effect COVID-19 has had on Alaska Native persons (*4*–*6*), who account for 89% of the population in the Yukon-Kuskokwim Delta region (*3*).

The findings in this report are subject to at least four limitations. First, the study is ecologic with no control region, therefore, a causal relationship cannot be inferred between introduction of BinaxNOW and the observed reduction in numbers of cases. The extent to which other factors, such as enhanced community mitigation efforts, might have contributed to the steep decline in case counts in November is unclear. Second, antigen tests have a higher false negative rate for the presence of virus than most molecular diagnostic tests (*7*). This might have led to lower detection of cases, particularly among asymptomatic persons, because symptomatic persons who received a negative test result were typically retested in 3 days. Moreover, one of the unanticipated challenges of relying on rapid point-of-care antigen testing was that persons frequently mistook a negative antigen test result as an indication that they no longer needed to isolate until serial repeat testing was completed. Third, not all persons who had a negative antigen test result were retested, which might have decreased the false-negative error rate. Finally, the definition of turnaround time could not practically include the time from obtaining the laboratory result to the point of interaction with the person tested, and variation in responsiveness to instructions to isolate could affect the conclusion.

High vaccination coverage is necessary to reduce COVID-19–related morbidity and mortality. The findings from this study indicate that rapid point-of-care antigen testing can be a valuable tool in reducing test turnaround times in rural communities where local access to laboratory-based NAAT is not readily available. Quickly providing infected persons with a positive test result could help facilitate actions to reduce further SARS-CoV-2 transmission, including prompt isolation, contact tracing, appropriate quarantine of close contacts, and prompt initiation of treatment when warranted.

**Summary**

**What is already known about this topic?**

Until the widespread availability of rapid point-of-care COVID-19 testing, one of the primary challenges in rural Alaska was slow turnaround times for SARS-CoV-2 laboratory-based nucleic acid amplification test results.

**What is added by this report?**

Introduction of rapid, point-of-care antigen testing in Alaska’s remote Yukon-Kuskokwim Delta region was followed by a more than threefold reduction in daily SARS-CoV-2 case rates during approximately 1 month before the introduction of COVID-19 vaccination.

**What are the implications for public health practice?**

Rapid point-of-care antigen testing shortens the turn-around time and might be a valuable tool in reducing transmission of SARS-CoV-2 in rural communities by facilitating rapid isolation and quarantine.

## Footnotes

* Symptomatic persons were typically identified through clinic visits or through questionnaires used during mass testing events in villages experiencing outbreaks. Persons who received positive test results were instructed to isolate and were interviewed by a contact tracer. Symptomatic persons and selected close contacts of persons with confirmed COVID-19 who had a high pretest probability of SARS-CoV-2 infection but initially received a negative test result were retested in 3 days.

† 45 C.F.R. part 46.102(l)(2); 21 C.F.R. part 56; 42 U.S.C. Sect. 241(d); 5 U.S.C. Sect. 552a; 44 U.S.C. Sect. 3501 et seq.
